# Decellularized Tissues for Wound Healing: Towards Closing the Gap Between Scaffold Design and Effective Extracellular Matrix Remodeling

**DOI:** 10.3389/fbioe.2022.821852

**Published:** 2022-02-16

**Authors:** Víctor Alfonso Solarte David, Viviana Raquel Güiza-Argüello, Martha L. Arango-Rodríguez, Claudia L. Sossa, Silvia M. Becerra-Bayona

**Affiliations:** ^1^ Program of Medicine, Faculty of Health Sciences, Universidad Autónoma de Bucaramanga, Bucaramanga, Colombia; ^2^ Program of Biomedical Engineering, Faculty of Engineering, Universidad Autónoma de Bucaramanga, Bucaramanga, Colombia; ^3^ Metallurgical Engineering and Materials Science Department, Faculty of Physicochemical Engineering, Universidad Industrial de Santander, Bucaramanga, Colombia; ^4^ Multi-tissue Bank and Advanced Therapy Center, Fundación Oftalmológica de Santander, Clínica Carlos Ardila Lulle, Floridablanca, Colombia

**Keywords:** acellular extracellular matrix, wound healing, extracellular matrix proteins, extracellular matrix remodeling, tissue scaffolds, extracellular matrix (ECM)

## Abstract

The absence or damage of a tissue is the main cause of most acute or chronic diseases and are one of the appealing challenges that novel therapeutic alternatives have, in order to recover lost functions through tissue regeneration. Chronic cutaneous lesions are the most frequent cause of wounds, being a massive area of regenerative medicine and tissue engineering to have efforts to develop new bioactive medical products that not only allow an appropriate and rapid healing, but also avoid severe complications such as bacterial infections. In tissue repair and regeneration processes, there are several overlapping stages that involve the synergy of cells, the extracellular matrix (ECM) and biomolecules, which coordinate processes of ECM remodeling as well as cell proliferation and differentiation. Although these three components play a crucial role in the wound healing process, the ECM has the function of acting as a biological platform to permit the correct interaction between them. In particular, ECM is a mixture of crosslinked proteins that contain bioactive domains that cells recognize in order to promote migration, proliferation and differentiation. Currently, tissue engineering has employed several synthetic polymers to design bioactive scaffolds to mimic the native ECM, by combining biopolymers with growth factors including collagen and fibrinogen. Among these, decellularized tissues have been proposed as an alternative for reconstructing cutaneous lesions since they maintain the complex protein conformation, providing the required functional domains for cell differentiation. In this review, we present an in-depth discussion of different natural matrixes recently employed for designing novel therapeutic alternatives for treating cutaneous injuries, and overview some future perspectives in this area.

## Introduction

Tissue engineering involves the use of stem cells, scaffolds and biochemical cues that elicit the cell response required to repair or regenerate specific tissues. Stem cell differentiation is regulated by different biomolecules and mechanical signals that are present in the extracellular microenvironment, known as the extracellular matrix (ECM), including transcription factors and structural proteins produced by cells (Cho et al., 2019). This ECM is being constantly remodeled, depending on the stage of stem cell differentiation: commitment, determination, or maturation. Also, the proteins, glycosaminoglycans and proteoglycans found in the ECM, along with ECM stiffness, activate the intracellular signaling pathways that control cell fate (Hoshiba et al., 2016). Therefore, gaining more insight into the intricate ECM dynamics underlying stem cell behavior may unlock the door to novel and effective strategies to induce specific tissue regeneration.

Human skin is one of the most thoroughly studied organs, due to its vital role as a protective barrier, as well as its unique regenerative properties. In fact, upon injury, distinct and coordinated cell processes start to develop to support skin restoration (Nelson and Bissell, 2006). Particularly, normal wound healing includes four stages: inflammation, re-epithelialization, angiogenesis, and ECM remodeling (Becerra-Bayona et al., 2020). In each of these phases, several types of cells and multiple ECM components and cytokines are essential to properly restore the epidermis and dermis layers (Kim et al., 2018; Tan et al., 2019). For instance, fibroblasts have a relevant role during ECM remodeling by producing collagen and other ECM proteins that allow for dermis maturation (de Mayo et al., 2017). Also, homeostasis and adequate levels of specific proteins are required in each of the wound healing stages to avoid fibrosis and scarring (Dai et al., 2020). When scarring occurs, the conformation of the new skin may differ from that of the native tissue. Similarly, chronic wounds are the consequence of an abnormal healing process in which the healing stages did not take place in the correct order, and one or more stages were not completed. Thus, for cases in which normal skin restoration has been disrupted, several treatment options have been developed to promote and accelerate wound closure (Tracy et al., 2016).

In this sense, several tissue engineering approaches have focused on understanding the effect of scaffold biochemical properties as well as scaffold structure (at the micro and nano levels) on stem cell behavior, towards promoting fast wound closure and, thus, functional skin regeneration (Hoshiba et al., 2016). Specifically, scaffolds provide a microenvironment that resembles that of native tissue (epidermis or dermis), which favors cell migration, angiogenesis, proliferation, differentiation, and ECM production during wound repair (Xu et al., 2021). Nonetheless, advanced alternatives are required when damage to the dermis occurs, since current clinical techniques such as antibiotics, debridement, negative pressure devices, and dressings (de Mayo et al., 2017), fail to provide an effective solution. In this scenario, wound closure is more difficult to achieve, given that the repair process takes place in an asynchronous or discontinuous pattern. This results in ECM instability in the chronic wound, because of the presence of a permanent inflammatory environment and an imbalance between proteolytic enzymes and protease inhibitors, yielding high amounts of matrix metalloproteinases (MMPs) that destroy the regenerated ECM ((Choi et al., 2013). In this context, different skin substitutes for ECM replacement are commercially offered, which are fabricated from autologous, allogenic, and xenogeneic sources (Table 1) (Valacchi et al., 2012; Tracy et al., 2016; Goodarzi et al., 2018; Dai et al., 2020). Regarding autografts, their use reduces rejection outcomes, but harvesting is limited and may cause pain and infection in the donor site. For their part, allografts and xenografts can overcome these limitations, but they may lead to immunological rejection and disease transmission (Song et al., 2017)). Consequently, the need to advance existing skin substitutes is compelling, towards developing novel alternatives for engineering biomimetic wound dressings that not only promote skin regeneration through ECM production and cell attachment, but also maintain a moist environment while avoiding inflammatory responses (Kirsner et al., 2015; Kim et al., 2018).

**TABLE 1:** Summary of several commercially available treatments for wound regeneration.

Name	Type	Source	Composition	Components relevant to wound healing	References
Epicel	Autograft	Human keratinocytes	Cultured keratinocytes in a fibrin mesh	Not reported	Nathoo et al. (2014)
CellSpray	Autograft	Human keratinocytes	Keratinocytes dispersed in aerosol	Not reported	Nathoo et al. (2014)
Epidex	Autograft	Scalp hair follicles	Cultured keratinocytes on a silicon membrane	Not reported	Dai et al. (2020)
Epifix	Allograft	Human amion and chorion membrane	Basement membrane that contains epithelial cells	Collagen I, collagen III, decorin	Dai et al. (2020)
Alloderm	Allograft	Human cadaveric skin	Decellularized cryopreserved basement membrane	Collagen I, elastin	Nathoo et al. (2014)
dCELL	Allograft	Human cadaveric dermis	Decellularized dermis cryopreserved with glycerol	Collagen I, collagen III, decorin	Elliott et al. (2015)
TransCyte	Allograft	Human newborn fibroblasts	Bilayer graft: 1) nylon mesh coated with porcine dermal collagen, and 2) silicon membrane seeded with fibroblasts	Collagen I, collagen III, collagen V, fibronectin, versican, decorin, tenascin	Noordenbos et al. (1999)
Dermagraft	Allograft	Human living neonatal foreskin fibroblasts	Cultured fibroblasts on a mesh made of polyglycolic acid	Collagen I, collagen III, collagen IV, elastin, decorin	Dai et al. (2020)
StrataGraft	Allograft	Neonatal keratinocytes	Neonatal keratinocytes used to produce a biologically functional stratified epidermis	Collagen I	Schurr et al. (2009)
Apligraft	Allograft	Bovine collagen and neonatal fibroblasts and keratinocytes	Layer of 1) collagen gel with neonatal fibroblast (dermis), and 2) neonatal keratinocytes (epidermis)	Collagen I	Trent and Kirsner, (1998)
OrCel	Allograft	Human neonatal foreskin fibroblasts and keratinocytes	Neonatal foreskin fibroblasts and keratinocytes cultured in bovine collagen sponge	Collagen I	Windsor et al. (2009)
Matriderm	Xenograft	Ligamentum nuchae of cattle	Lyophilized dermis coated with elastin hydrolysate	Collagen I, collagen III, collagen V, elastin	Halim et al. (2010)
Biobrane	Xenograft	Porcine collagen	Bilaminar nylon mesh filled with 1) collagen I, and 2) thin silicone lamina	Collagen I	Alrubaiy and Al-Rubaiy, (2009)
OASIS	Xenograft	Porcine jejunum submucosa	Lyophilized small intestine submucosa	Collagen I, Collagen III, Collagen IV, Collagen VI, fibronectin, elastin, hyaluronan, chondroitin sulfate, decorin	Yeh et al. (2017)
Integra	Xenograft	Bovine collagen and shark chondroitin sulfate	Layer of 1) collagen I from tendon, 2) chondroitine-6-sulfate, and 3) a silicone pseudoepidermis	Collagen I, chondroitin sulfate	Philandrianos et al. (2012)
Nevelia	Xenograft	Bovine collagen	Three-dimensional porous matrix composed of 1) collagen and 2) a semi-permeable silicone membrane	Collagen I	Nicoletti et al. (2018)
Permacol	Xenograft	Porcine dermal tissue matrix	Collagen and elastin crosslinked by diisocyante	Collagen I	Hsu et al. (2009)
Pri-matrix	Xenograft	Fetal bovine dermis	Acellular dermal matrix	Collagen I, collagen III	Sutjarittangtham et al. (2014)

Over the last few decades, decellularized ECM (dECM) obtained from various human and animal tissues has been employed for burn treatment and surgical reconstruction (Table 1) (Wainwright, 1995). A growing body of research indicates that dECM guides wound healing progression by coordinating cell phenotype and ECM protein production by, for example, modulating the pro-inflammatory and anti-inflammatory macrophage phenotypes (M1 and M2, respectively), which release cytokines for cell homing and induce tissue remodeling, respectively (Mosser and Edwards, 2008; Rodero and Khosrotehrani, 2010). In this context, Dziki et al. reported that decellularized tissue from small intestinal mucosa, urinary bladder, brain, esophagus, and colon generated M2-macrophage phenotypes, while decellularized dermis promoted M1-macrophage phenotype. On the other hand, the authors showed that ECM derived from skeletal muscle and liver had no remarkable effect on macrophage response (Dziki et al., 2017), demonstrating that dECM effectiveness on acute and chronic wounds depends on its physical, biological, and chemical features, which may differ based on the tissue source and decellularization technique that are used (Milan et al., 2020). Similarly, other authors have reported that dECM influences fibroblast and myofibroblast differentiation by modulating collagen production and induces angiogenesis by promoting endothelial cell migration (Wang et al., 2021a). Therefore, the main goal of this review was to provide a comprehensive analysis of the natural ECM remodeling processes that take place during wound healing, highlighting the importance of the intricate interplay among several ECM elements, without which proper skin regeneration would not be possible. Thus, dECM and dECM-derived scaffolds are discussed as promising tools for the design of skin substitutes that more accurately resemble the biochemical and mechanical complexity of native ECM.

## Skin Structure and Wound Healing Process

Skin is a vital and complex organ, due to its multifaceted role as a temperature regulator as well as a physical, chemical, and microbiological barrier that protects the host from external harm (Kanitakis, 2002). In addition, the intricate network of immune cells that resides within this tissue is crucial for host defense and tissue homeostasis (Nguyen and Soulika, 2019). Skin is made up of three layers: epidermis, dermis, and hypodermis (subcutaneous layer). The epidermis is the outermost layer of skin that: 1) prevents microbes, fungi, viruses, toxins, allergens, and irritants from entering the body, 2) absorbs ultraviolet radiation, 3) reduces damage by mechanical forces, and 4) regulates moisture content in the body as well as ion and metabolite loss (Eckhart and Zeeuwen, 2018). Keratinocytes are the most abundant cells in the epidermis (approximately 90%), defining its structure and forming the cornified cell layers that are in direct contact with the environment. The rest of the epidermis is made up of melanocytes, Langerhans cells, and Merkel cells, being the entire cell population distributed throughout several layers, depending on the morphology and biological functions of keratinocytes (Yousef et al., 2021).

The dermis is the mesenchymal component of skin, which is separated from the epidermis by the basement membrane and represents the inner layer of skin between the epidermis and the hypodermis (Rippa et al., 2019). It accounts for approximately 90% of the weight of skin, constituting the foundation of this organ (Nourian Dehkordi et al., 2019). The dermis is a connective tissue composed of ECM, vascular endothelial cells, and fibroblasts, along with adipose glands, sweat glands, hair follicles, blood vessels, and nerve endings. Fibroblasts are the main dermal cell population, and their primary role is to produce collagen and elastin, which confer mechanical strength and elasticity to the skin. Also, the dermis comprises two structurally different layers: the papillary and reticular layers. The papillary layer is located closer to the skin surface and is organized into cords, which are called dermal papillae and contain nerve endings and microvascular vessels that are fundamental for nourishment and innervation. For its part, the reticular dermis is separated from the papillary layer by a subpapillary plexus (Rippa et al., 2019). Finally, the hypodermis is the deepest layer of the skin, consisting of loose connective tissue, fat-storing cells, blood vessels, and nerves. This tissue is especially rich in proteoglycans and glycosaminoglycans that support water retention within the tissue (Wong et al., 2016).

### Normal Wound Repair Process

**FIGURE 1:**
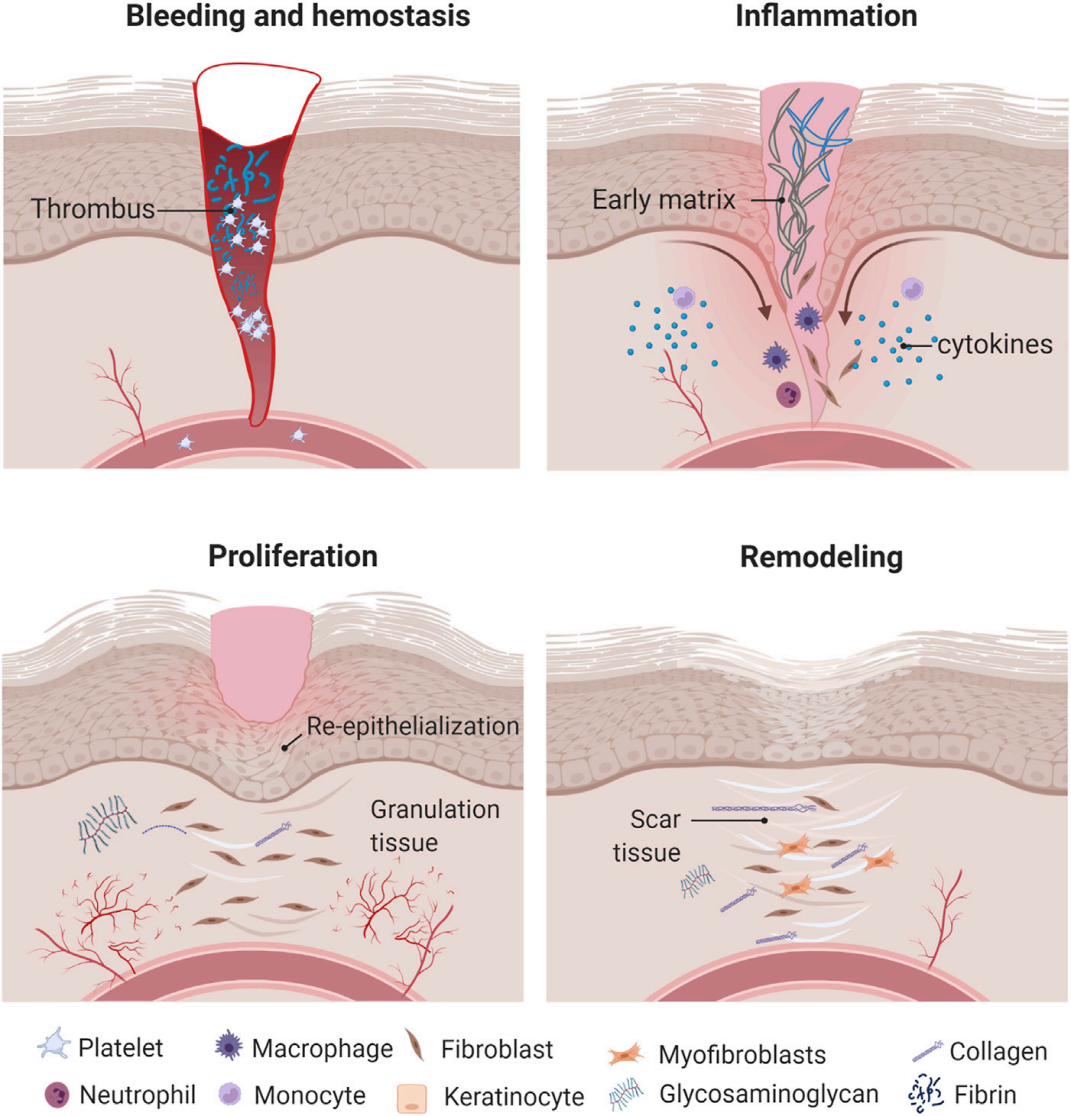
Wound healing process. Once the wound is formed, inflammatory cells produce different cytokines and growth factors that promote cell migration and proliferation as well as ECM formation and remodeling. Created with BioRender.com.

Wound healing is a complex and highly regulated biological process that is critical for maintaining the barrier function of skin. It includes four stages that are interrelated and suggest a continuum process: hemostasis, inflammation, proliferation, and remodeling (Figure 1). These involve the delivery of different cell types, chemokines, cytokines, growth factors, matrix molecules, and nutrients to the wound site.

#### Hemostasis Phase

The first response to a skin wound is vasoconstriction of the injured blood vessels to stop the bleeding; this phase lasts 2–3 h. Next, primary and secondary hemostasis occur via two concurrent and mechanistically intertwined pathways (Chaudhry et al., 2021). Primary hemostasis includes platelet adhesion, activation and further aggregation caused by exposure of subendothelial matrix proteins (collagen, von Willebrand factor, and fibronectin), which ultimately results in the formation of a plug at the damaged site. Multiple receptors on the surface of platelets are involved in these adhesive interactions (e.g., GPIb-IX-V, GPVI, and G protein coupled receptors), which are targeted by multiple adhesive proteins (Gale, 2011).

Moreover, secondary hemostasis refers to the activation of the coagulation cascade through which soluble fibrinogen is converted to insoluble strands that make up the fibrin mesh. The platelet plug and the fibrin mesh combine to form the thrombus, which stops the bleeding, release complements and growth factors, and provide a temporary scaffold for cell infiltration during wound healing (Gale, 2011). Secondary hemostasis includes two main coagulation pathways, extrinsic and intrinsic. The extrinsic coagulation pathway occurs when the vascular system is injured and the blood is exposed to extravascular tissues, which are rich in tissue factor (TF), a cofactor for the serine protease factor VIIa (Kirchhofer and Nemerson, 1996). The TF and factor VIIa complex activate factor X and factor IX. Factor IXa also activates factor X, in the presence of its cofactor, factor VIIIa. Likewise, in the presence of its cofactor, factor Va, factor Xa then activates prothrombin to generate thrombin (Dahlbäck, 2000). Thrombin is the central serine protease in the coagulation cascade, playing a critical role in several reactions (Lane et al., 2005). For instance, thrombin cleaves fibrinogen to generate insoluble fibrin, triggers platelets via cleavage of PAR1 and PAR4 (Kahn et al., 1998), and is also responsible for positive feedback activation of coagulation, which is essential for clot propagation. Furthermore, the intrinsic coagulation pathway takes place when thrombin activates cofactors VIII and V, and factor XI, which then activates factor IX (Lane et al., 2005).

Similar to the coagulation cascade, fibrinolysis is tightly controlled by a series of cofactors, inhibitors and receptors (Collen and Lijnen, 1995). The role of the fibrinolytic system is to dissolve blood clots during wound healing and to prevent blood clot formation in healthy blood vessels (Gale, 2011). The fibrinolytic system is primarily composed of three serine proteases that are present as zymogens in the blood. Plasmin is the primary fibrinolysin and is activated from plasminogen by either of two primary serine proteases: tissue-type plasminogen activator (tPA), or urokinase-type plasminogen activator (uPA) (Chapin and Hajjar, 2015). Whereas tPA is synthesized and released by endothelial cells, uPA is produced by monocytes, macrophages, and urinary epithelium. Both activators have an exceedingly short circulation half-life (4–8 min) due to the presence of high concentrations of specific inhibitors, or serpins, which are also important to prevent excess amounts of unregulated activator (Travis and Salvesen, 1983). The most important serpins in fibrinolysis are plasminogen activator inhibitor-1 (PAI-1), plasminogen activator inhibitor-2 (PAI-2), and α2-antiplasmin (A2AP). When plasmin is bound to fibrin, however, it is protected from A2AP inhibition, allowing for fibrinolysis to proceed (Schneider and Nesheim, 2004). Other non-serpin plasmin inhibitors include α2-macroglobulin, C1-esterase inhibitor, and members of the contact pathway of the coagulation cascade, which also play minor roles in plasmin inhibition. Thrombin-activatable fibrinolysis inhibitor (TAFI) is a non-serpin attenuator that is activated by thrombomodulin-associated thrombin. TAFI is a carboxypeptidase that removes C-terminal lysine and arginine residues on fibrin, thereby decreasing the number of available plasminogen binding sites, slowing plasmin generation, and stabilizing clots (Broze and Higuchi, 1996).

#### Inflammation Phase

The inflammatory phase mainly involves the activation of the innate immune system so that neutrophils and monocytes rapidly migrate to the injury site. This phase in wound healing starts shortly after hemostasis is achieved and can last from hours to days in acute wounds, while in chronic wounds it can last for weeks or even months, because of the effects of the underlying disease. The primary goal of the inflammatory phase is to clear pathogens as well as foreign material from the wound and to contain the damage to a localized area (Wynn and Barron, 2010). Vascular permeability increases with vasodilation, allowing neutrophils and monocytes to identify the wound site (Han and Ceilley, 2017). A complex interplay of cytokines recruit neutrophils and monocytes to the wound area, culminating in monocyte conversion to macrophages, often thought of as the master regulator of this inflammatory phase of wound healing. Macrophages not only engulf and digest tissue debris and remaining neutrophils, but also secrete growth factors and cytokines that promote tissue proliferation and cell migration (Wallace et al., 2021).

#### Proliferation Phase

About 3 days after injury has occurred, the proliferative phase centers around fibroblasts and their production of collagen as well as the ground substance that will form the foundation of the tissue scaffold in the wound area. Meanwhile, endothelial cells initiate a rapid growth phase and angiogenesis occurs within the granulation tissue, creating a rich vascular network for the healing zone. The proliferative phase can last several weeks and is characterized by the formation of granulation tissue, re-epithelialization, and neovascularization. During this phase, fibroblasts start to lay down new collagen and glycosaminoglycans, which form the core of the wound and help stabilize it. Then, re-epithelialization starts with the migration of cells from the wound periphery and adjacent edges. Initially, only a thin superficial layer of epithelial cells is laid down, but a thicker and more durable layer of cells will bridge the wound over time (Rodrigues et al., 2019).

On the other hand, endothelial cell and fibroblast proliferation and migration support angiogenesis and new ECM formation, respectively. As the new ECM is reconstructed, the old matrix is degraded by proteases (MMPs). MMPs promote autolytic debridement and cell migration into wounds. The level of MMPs in wounds increases after tissue damage and decreases with remission of inflammation. Epithelial cells migrate from the edge of the wound, initiating epithelialization. Keratinocyte differentiation helps restore the barrier function of the epidermis. Next, neovascularization occurs through angiogenesis and vasculogenesis, which refer to the formation of new blood vessels from existing vessels and endothelial progenitor cells (EPCs), respectively. Once collagen fibers have been laid down on the fibrin framework, the wound starts to mature and contract, being the latter process supported by the continued deposition of fibroblasts and myofibroblasts (Wallace et al., 2021).

#### Remodeling Phase

The maturation or remodeling phase starts around week 3 and can last up to 12 months. Eventually, an eschar (scab) forms on the surface of the wound (Monavarian et al., 2019). During this phase, the ECM is constantly being reconstructed by myofibroblasts, and wound contraction is induced by the dense network of collagen microfilaments. At the same time, new components are secreted to increase matrix density and stability (Landén et al., 2016). Furthermore, the proportion of different types of collagens begins to change, as type I collagen proportion increases (80–90%), while collagen III proportion decreases (10–20%). The excess collagen degrades, and wound contraction also begins to peak at around week 3. Finally, after approximately 2–3 weeks, the wound transitions to a remodeling or maturation stage, in which collagen I levels are fully restored and the wound tissue matures, resulting in full cross-linking and restoration of a somewhat normal structure (Xue and Jackson, 2015). In addition to this, apoptosis reduces the density of myofibroblasts, creating room for fibroblasts, which further increases ECM mechanical resistance (Landén et al., 2016). Wound contraction occurs to a much greater extent during secondary healing, relative to the primary healing phase. The maximal tensile strength of the wound area is achieved after about 11–14 weeks, although it never fully reaches its normal, pre-injury mechanical state (only about 80% of the original tissue’s tensile strength is recovered) (Bowden et al., 2016).

### Chronic Wound Healing

**FIGURE 2:**
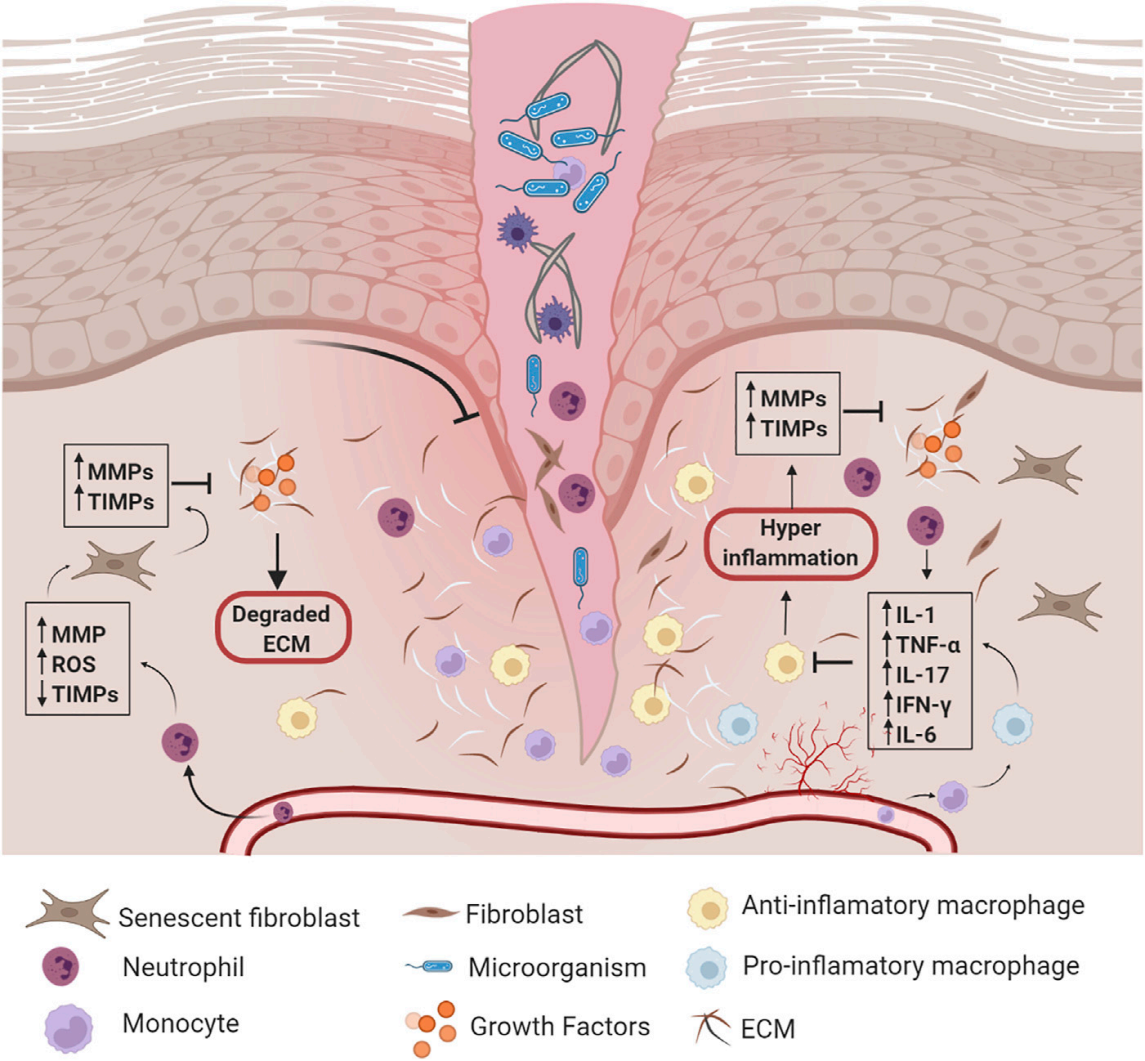
Chronic wound healing. Once the wound is formed, synergy among hemostasis, inflammation, proliferation, and remodeling phases are disrupted by different underlying pathological mechanisms. *Abbreviations: MMPs: Matrix Metalloproteinases, TIMPs: Tissue Inhibitors of Metalloproteinases, ROS: Reactive Oxygen Species, ECM: extracellular matrix, IL-1: Interleukin-1, TNF-α: Tumor Necrosis Factor-Alpha, IL-17: Interleukin-17, IFN-γ: Interferon Gamma, IL-6: Interleukin-6.* Created with BioRender.com.

Chronic wounds develop when normal wound healing is delayed or disrupted by different underlying pathological mechanisms, such as continuous inflammation, persistent infections, and necrosis (Raziyeva et al., 2021). Besides, chronic wounds are associated with the impairment of the following processes: growth factor production, angiogenic response, macrophage differentiation, collagen production, epidermal barrier function, granulation tissue formation, keratinocyte and fibroblast migration/proliferation, and bone healing (osteomyelitis has been reported as a predictor of inadequate wound healing and amputation (Burgess et al., 2021)). Compared with normal wound healing, chronic wounds are characterized by changes in the healing phases, as follows: 1) during hemostasis, the hypercoagulability and the decrease in fibrinolysis are altered (Erem et al., 2005); 2) during the inflammatory stage, there is an imbalance of inflammatory cytokines such as interleukin (IL)-1 (IL1), IL6, tumor necrosis factor-alpha (TNF-α), and interferon gamma (IFN-γ), as well as several growth factors, such as platelet derived growth factor (PDGF), epidermal growth factor (EGF), and insulin-like growth factor 1 (IGF-1) (Pradhan et al., 2009); 3) fibroblast and keratinocyte migration and proliferation are diminished; and 4) an imbalance between the accumulation of ECM components and their remodeling by MMPs (Brem and Tomic-Canic, 2007) (Figure 2). Decreased cell migration results in deficient re-epithelialization of the chronic wound (Chen et al., 2016), which contributes to defective wound closure and decreased angiogenesis. All of these alterations affect the remodeling phase of wound healing (Santoro and Gaudino, 2005).

## The Role of Extracellular Matrix in Wound Healing

Wound healing begins with a temporary ECM, which is mainly composed of fibrin, plasma fibronectin (FN), vitronectin, and platelets (Chester and Brown, 2017). FN is synthesized as a soluble form by hepatocytes, and it plays an important role in the early phase of wound healing (hemostasis), as it binds to platelets and fibrin, thereby increasing the strength of the fibrin clot (To and Midwood, 2011). As early as 2 days after injury, macrophages increase FN mRNA expression; similarly, granulation tissue fibroblasts begin to express FN proteins at about 7 days after injury (Barker and Engler, 2017). FN is a multidomain glycoprotein with a self-assembled domain that allows FN dimers to interact, producing a three-dimensional (3D) matrix through a process termed fibrillogenesis (Mao and Schwarzbauer, 2005). Given that FN has different binding conditions to different matrix proteins such as heparin, syndecan, and proteoglycans, it facilitates gradual protein and cell assembly within the matrix as healing progresses (Mao and Schwarzbauer, 2005). To mediate cell assembly, FN dimers bind to transmembrane integrin receptors, such as α5β1, while integrins link FN to the actin cytoskeleton through cytoplasmic domain interactions with cytoskeletal proteins (Wu, 1997; Wierzbicka-Patynowski and Schwarzbauer, 2003).

Another important protein in the ECM remodeling is tenascin-C, which is primarily produced by keratinocytes. This matrix protein binds to many extracellular elements, such as soluble factors, matrix proteins, and cells (through interactions with surface receptors) (Midwood et al., 2016). The expression of tenascin is a hallmark of inflammation, and it is present in areas with increased immune cell infiltration during the acute inflammation phase. Along with polymorphonuclear lymphocytes, tenascin is located at the inflamed human dermis, stimulating migration and activation of immune cells (Midwood et al., 2016). Tenascin-C stimulates the production of pro-inflammatory cytokines (TNF-α, IL6, and IL8) in macrophages and fibroblasts by activation of Toll-like receptor 4-mediated signaling pathways (Midwood et al., 2009). Moreover, tenascin-C is also found in new granulation tissue, promoting fibroblast migration and tissue reconstruction; however, its expression decreases at the end of the healing process, indicating that its activity is temporary (Mackie et al., 1988; Midwood et al., 2016).

As wound healing progresses, the role of collagen becomes more important during re-epithelialization, granulation tissue formation, and neovascularization. When granulation tissue formation starts, fibroblasts migrate and produce fibrillar collagen, which ends up replacing most of the temporary fibrin matrix (Bainbridge, 2013). Collagen I can induce keratinocyte migration, which initiates re-epithelialization, as well as keratinocyte secretion of MMPs that allow matrix remodeling (Guo et al., 1990). The appearance of collagen coincides with the early onset of vessel formation, since, for instance, collagens I and III contain von Willebrand factor (vWF) domains that have been shown to regulate bone morphogenetic protein, which promotes blood vessel morphogenesis (Manon-Jensen et al., 2016). Collagen V also has vWF domains, but in this case, this collagen has shown a relevant role in modulating fibroblast migration-proliferation and tissue contraction (Berendsen et al., 2006; Manon-Jensen et al., 2016). During the initial stages of healing, other types of collagens are also found, such as type VI, which has been shown to modulate macrophage adhesion in the healing site (Schnoor et al., 2008), as well as support ECM architecture during ECM deposition (Oono et al., 1993).

On the other hand, collagen VII, plays a role in fibroblast and keratinocyte migration, as its absence has been demonstrated to delay granulation tissue formation and re-epithelialization (Nyström et al., 2013). Collagen VII is the major component of anchoring fibrils at the dermal-epidermal junction, and thus, when it is not present in normal amounts, the skin’s resistance to frictional forces is reduced, which can be evidenced as skin blistering (Kiritsi and Nyström, 2018). Moreover, other types of collagens, such as type IV, are the structural foundation for all basement membranes and can be detected in the newly formed tissue after 8–21 days (Cosgrove and Liu, 2017). Collagen IV and laminin self-assemble into two independent supramolecular networks that are linked to nidogen and perlecan to form a morphological discernable basement membrane basal lamina (Mak and Mei, 2017).

Moreover, transmembrane collagen XVII is a highly dynamic modulator of *in vivo* proliferation and motility in activated keratinocytes during epidermal regeneration and is also involved in the anchorage of the epidermis to the underlying basement membrane (Löffek et al., 2014). Collagen XVII is an important part of the hemidesmosomal transmembrane component, modulating integrin-dependent keratinocyte migration via PI3K/Rac1 signaling, through stabilization of the lamellipodia at the leading edge of re-epithelializing wounds (Löffek et al., 2014). Collagen XVII is abundantly secreted in keratinocytes during re-epithelialization of acute wounds and its absence appears to lead to altered cell motility in the wound (Löffek et al., 2014). On the other hand, overexpression of collagen XVIII negatively affects wound healing, since it slows skin repair, inhibits vascularization, as well as decreases myofibroblast density (Seppinen et al., 2008). Thus, collagen regulation is essential for normal wound healing (Rousselle et al., 2019).

Furthermore, as wound healing progresses, other proteins become involved in the process. That is the case of laminin (LM). This matrix protein is a major constituent of the basement membrane separating the epithelium, mesothelium, and endothelium from connective tissue, and although there are 15 different types of LMs, only some have been fully identified during the healing process, such as LM511, LM521 and LM332. At the beginning of the epithelialization process, within hours of injury, LM332 is expressed by epidermal keratinocytes, inducing their migration. It should be noted that LM332 appears to be the first base membrane component laid down onto the wound bed, as its early expression precedes that of all other ECM components (Laplante et al., 2001). A LM322 domain such as LG45 plays a major role in regulating mechanisms underlying keratinocyte and ECM remodeling during wound repair. LG45 induces expression of the MMP-9 pro-enzyme, which together with MMP-14, exert proteolytic activity within epithelial podosomes, allowing cell scaffolding (Michopoulou et al., 2020). Moreover, LG45-derived peptides have antimicrobial activity and chemotactic activity in mononuclear cells, suggesting that this domain may also participate in host defense (Senyürek et al., 2014). On the other hand, LM511 and LM521 have been found to be related to epidermal regeneration; their expression and deposition on the base membrane increase after re-epithelialization is completed, and they have been suggested to be key factors in promoting keratinocyte differentiation as well (Pouliot et al., 2002; Sugawara et al., 2008).

In addition to this, during late stages of healing, perlecan or heparan sulfate proteoglycan 2 (HSPG2), is an essential protein for basement membrane assembly. It has a connective function, as it aids in creating molecular bridges that fit with cellular interactions (Hohenester and Yurchenco, 2013), orchestrating the binding and signaling of mitogens and morphogens to cells in a temporal and dynamic fashion (Lord et al., 2018). Perlecan acts as a reservoir for heparin-binding growth factors that accelerate keratinocyte migration and skin wound healing (Shirakata et al., 2005). Among other features, the degradation of perlecan during wound healing allows for a rapid introduction of mitogens and trophic factors that modulate the regenerative processes (Lord et al., 2018). Also, perlecan is located along with LM332 at the wound margin of full thickness wounds, which appears to indicate that it is closely related to base membrane remodeling (Yurchenco, 2015); thus, it has been suggested that perlecan plays a role in keratinocyte survival and terminal differentiation (Sher et al., 2006; Whitelock et al., 2008). Nidogens are another family of proteins involved in base membrane restoration, as they work as connecting bridges for other proteins, such as LM, heparan sulfate, proteoglycan and collagen IV (Aumailley et al., 1993), which provide the mechanical reinforcement required to maintain the cytoarchitecture of the skin (Breitkreutz et al., 2009).

On the other hand, structural proteins are abundantly expressed in response to injury. This is the case of thrombospondin (TSP), a protein produced by fibroblasts that can also be found in platelet α-granules (Kyriakides and Maclauchlan, 2009; Rousselle et al., 2019). TSP1 and TSP2 are crucial for developmental angiogenesis; however, while TSP1 is early induced in wound healing and participates in the activation of latent transforming growth factor beta 1 (TGF-β1), TSP2 expression occurs after resolution of the inflammatory phase and during granulation tissue remodeling. Moreover, TSP2 plays a key role in the controlled release of vascular endothelial growth factor (VEGF), as well as MMP-2 and MMP-9 during ECM remodeling (Maclauchlan et al., 2009). Similarly, osteopontin is a structural chemokine-like protein that acts as part of an intracellular signaling complex, taking a role in the formation of fibrous tissue (Icer and Gezmen-Karadag, 2018). It behaves as a cytokine, for example, as fibroblast chemoattractant, and it appears to be essential for fibroblast deposition of ECM components and collagen (Fujisawa et al., 2020), thus, playing a synergistic role with TSP (Rousselle et al., 2019).

For its part, osteonectin, also known as secreted protein acidic and rich in cysteine (SPARC), is a protein secreted by various types of cells, such as fibroblasts, endothelial cells, and platelets (Bradshaw and Sage, 2001), and its expression reaches the highest level during remodeling of the dermal ECM. SPARC participates in the regulation of cell adhesion, proliferation and matrix turnover, modulating the activity of growth factors and ECM, sequestering or releasing these factors according to the dynamic remodeling of the matrix (Motamed, 1999). Also, its mechanism of action is given by interconnection of different matrix proteins, including thrombospondin 1, vitronectin, entactin/nidogen, fibrillar collagens (types I, II, III, and V), and collagen IV, which allow for matrix reticulation (Brekken and Sage, 2000). Specifically, SPARC binds to PDGF and VEGF and decreases their mitogenic potency, due in part by its abrogation of growth factor–receptor interaction (Yan and Sage, 1999). In addition to PDGF, VEGF, and bFGF (basic fibroblast growth factor), SPARC can also modulate the activity of TGF-β, as seen in recent studies with SPARC-null mesangial cells, which allows it to regulate cell migration and differentiation during epithelialization in wound healing (Brekken and Sage, 2000).

Once wound closure is achieved, keratinocytes undergo stratification and differentiation to restore the natural barrier provided by the skin. Nevertheless, successful skin regeneration relies on the coordination of growth factors, cytokines, and chemokines, which must act in synergy with cells to modulate their behavior through specific cell surface receptors or ECM proteins (Davison-Kotler et al., 2019). It is important to note here that, although we have discussed a set of relevant proteins, there are additional proteins that are similarly involved in wound healing regulation.

## Decellularized Extracellular Matrix

Tissue engineering and regenerative medicine involve the use of different synthetic and natural materials, which are frequently combined to produce composite scaffolds. In terms of natural materials, dECM from autologous or allogenic sources appears to be one of the most promising types of scaffolds (Porzionato et al., 2018), which can be used either alone or in combination with other biological components for preclinical and clinical applications. For implantation procedures, tissue decellularization is required to avoid the immune reaction and inflammation response that could be induced by the cells in the source tissue, which otherwise may cause implant rejection (Mendibil et al., 2020). ECM decellularization allows for the obtention of cell-free matrices with low immunogenicity, in which several bioactive components from the natural ECM are preserved, such as structural and specialized proteins, proteoglycans, collagen, elastin, and hyaluronic acid, as well as growth factors (Mendibil et al., 2020).

Decellularized ECM can be sourced from native organs from different species to provide the biological cues needed for cell homing (Badylak et al., 2009). Nevertheless, xenogeneic tissues may carry residual immunogenicity and be contaminated with biological agents. For these reasons, human allogeneic or autologous tissues are the ideal dECM source material, being also suitable for recellularization and implantation (Duda et al., 1999). In addition, human tissues/organs may be obtained from surgery or cadavers, and the resulting dECM may be crosslinked to improve its mechanical properties (Yang et al., 2020; Xu et al., 2021). In fact, the use of dECM from human tissues and organs such as cartilage (hyalin, articular, meniscal, laryngeal, tracheal, and nasoseptal), pericardium, blood vessels, corneas, bone, lung, and nerve have been previously reported (Tao et al., 2021).

In terms of decellularization techniques, a variety of approaches have been studied, which include chemical, physical, and biological treatments, as well as their combinations (Hoshiba et al., 2016; Crapo et al., 2011) (Table 2). Specifically, some common decellularization methods are: 1) chemical treatments (i.e., sodium dodecyl sulfate (SDS), sodium lauryl sulfate, ethylenedimine tetraacetic acid (EDTA), Triton X-100, and Tris-HCl); 2) enzymatic treatments, (DNases and RNases), which are frequently used and combined in multistep procedures, although some residual DNA may remain at the end of the treatment; 3) physical methods; 4) osmotic treatments; 5) tissue mechanical fragmentation (or pulverization); and 6) freeze/thaw cycles, which enable pore formation upon ice crystal formation within the tissue (Porzionato et al., 2018). The advantages and limitations of decellularization procedures have been previously discussed (Dussoyer et al., 2020), indicating that the abovementioned techniques can yield decellularized materials that maintain ECM composition levels similar to those of the source tissue, in addition to tissue architecture and 3D organization (Schenke-Layland and Nerem, 2011). In fact, acceptable dECM products can have residual amounts of DNA (up to 50 ng DNA per mg dry weight), preserve around 75% of the collagen component of the native ECM, and maintain natural ECM structure (Hsieh et al., 2020).

**TABLE 2:** Summary of several available methods for tissue decellularization.

Method	Main characteristics	Disadvantages	Decellularized tissues	Ref
*Chemical Methods*				
		(i) Collagen and glycosaminoglycans (GAGs) damage		(Ott et al., 2008)
Acid—base	Cell membrane solubilization	(ii) Insufficient cell removal	Rat heart	(Syed et al., 2014)
Peracetic acid	Disruption of cytoplasmic components and nucleic acids by utilizing charges	(iii) Increased ECM stiffness	Small intestine submucosa	Gilpin and Yang, (2017)
Ethylenediaminetetra-acetic acid (EDTA)		(iv) Decreases salt- and acid-soluble ECM proteins	Urinary bladder	
Reversible alkaline swelling		(v) Alters mechanical properties		
Triton X (100 or 200)	Disruption of lipid–lipid and lipid–protein unions, while leaving protein interactions	(i) Not recommended for ECM when lipids and GAGs are important components		(Wagner et al., 2014); Rieder et al. (2004)
	Very effective in some tissues	(ii) Limited potential by immunogenicity *in vivo*	Normal and emphysematous human lungs	
	Less damaging to tissue structures than ionic surfactants	(iii) Triton X-200 needs to be combined with a zwitterionic detergent to be effective	Porcine heart valves	
		(iv) Triton X-200 damages the matrix similar to SDS		
Sodium dodecyl sulfate (SDS)	Liquefaction of internal and external cell membranes	Tends to denaturalize proteins and induce nuclear and cytoplasmic waste in the remaining matrix (i) Cytotoxic: requires extensive washing steps	Rat forearm	(Yang et al., 2015); (Gilpin and Yang, 2017); Wang et al. (2010)
		(ii) Alters microstructure (i.e., collagen fibers)	Porcine tissues (cornea, myocardium, heart valve, small intestine, kidney)	
			Human vein, lungs and heart	
Witterionic, nondenaturing detergent, 3-[(3-cholamidopropyl) dimethylammonio]-1-propanesulfonate (CHAPS)	Properties of ionic and nonionic detergents	(i) Similar damage as Triton X-100	Human and porcine-derived lung tissues	(Gilpin and Yang, 2017); O'Neill et al. (2013)
	Maintenance of structural ECM proteins and ultrastructure	(ii) Remanent cytoplasmic proteins	Rat lungs	
Tributyl phosphate (TBP)	Destructor of protein structures	Variable results, leads to collagen degradation but keeping the mechanical properties	Equine flexor tendons, ligaments and articular cartilage	(Deeken et al., 2011); Elder et al. (2009)
	Disruption of protein–protein interactions			
Hypertonic and hypotonic solutions	Solutions with a higher/lower solute concentration than that in cells	High amount of cell waste in the remaining matrix	Bovine vessel nerve, small intestinal and submucosa	(Zhang et al., 2022); Kim et al. (2016)
	Cell lysis, cell dehydration and cell death because of their osmotic pressure			
*Enzymatic Methods*				
Trypsin	Digestion of membrane proteins leading to cell dead	(i) Can damage the proteins in the ECM, in particular laminin and GAGs	Porcine pulmonary valves and trachea	Giraldo-Gomez et al. (2016)
	Commonly used with EDTA	(ii) Breaks cell-matrix adhesions		
Pepsin	It targets peptide bonds	Causes high damage in the ECM proteins if left for long periods of time	Porcine lung and liver	(Pouliot et al., 2020); Coronado et al. (2017)
nuclease	Hydrolysis of DNA and RNA	Further cleaning and enzyme removal is required, as they may promote immune response	Bovine osteochondral plugs, human corneal limbus and porcine dermis	(Greco et al., 2015); Fermor et al. (2015)
*Physical Methods*				
Thermal shock (freeze-thaw cycle)	Disruption of tissue and organ cells	(i) The freeze-thaw cycle causes a small degradation in the structure of the ECM, due to the crystal shape that may damage the scaffold, with little effect on the mechanical properties of ECM	Tendon fragments (large), fibroblast sheets, lumbar vertebrae cells, kidneys, lungs and adipose tissues	(Rabbani et al., 2021); Zhao et al. (2019)
	Frozen water crystals occupy the volume inside the cell and cause the membrane to burst	(ii) The heat shock cycle alone is not capable of removing sensitive cellular components		
Force	Mechanical pressure can be enough to induce cell lysis	(i) Limited to tissues with hard structures, as it can damage the ECM structure	Liver, lung	Gilpin and Yang, (2017)
		(ii) The amount of required force must be precise since both the underlying structure and membrane attachment are vulnerable to any kind of direct mechanical stress		
Immersion and agitation	It is commonly used to facilitate chemical agent infiltration to induce cell lysis	Aggressive processes, such as sonication, can damage the ECM.	Submucosal substrate, laryngeal and intestine tissues	Keane et al. (2015)
	The immersion time and intensity of agitation depend on the thickness and density of the tissue			
Vacuum-assisted decellularization (VAD)	VAD would accelerate and improve the delivery and efficiency of detergents into the deepest parts of the tissue	It is not a decellularization method but a facilitator	Porcine tracheal specimen and fresh porcine costal cartilage	Alizadeh et al. (2019)
	Removal of detergents from a decellularized tissue is the other application of the VAD methodology			
Hydrostatic pressure (water is sprayed with pressure on the target tissue)	Application of high pressure (>600 MPa) to the tissue and induction of cell lysis	(i) Excessive pressure can damage the structure	Porcine retinal specimen, porcine artery, porcine meniscus and rat uterine	Rabbani et al. (2021)
		(ii) The formation of ice crystals caused by the presence of water may damage the ECM structure		
		(iii) Increasing the temperature during the process may suppress the creation of the ice crystals, but may increase the entropy and lead to the ECM vulnerability		
		(iv) Residue of DNA fragments		
		(v) Denatures ECM proteins		
Nonthermal irreversible electroporation	Microsecond electrical pulses are applied throughout a tissue, causing micropores in the cell membrane	The relatively small electrodes that limit the size of the tissue for decellularization	Carotid arteries of rat, liver of porcine and myocardial muscle tissue	Rabbani et al. (2021)
Ultrasonic waves (sonication)	High-power waves are capable of disrupting intermolecular bonds, disrupting the cell membrane, and removing its internal components	The physical phenomenon of cavitation during the process is unavoidable, but uncontrolled cavitation can severely damage the structure and mechanical properties of the tissue	Aortic tissues, small intestine, cartilage tissue and meniscus	Forouzesh et al. (2019)
Pressure gradient	Induction of a pressure gradient can help the enzyme-mediated decellularization method	To be determined	Embryonic veins, tendon, and aortic tissue	Sierad et al. (2015)
Supercritical fluid	Removal of cell debris. It is used in combination with detergents	To be determined	Porcine pericardium, aorta and retinal tissues	Guler et al. (2017)
	Reduction of the detrimental effect on the ECM mechanical properties			
Perfusion	The organ is completely separated from its main blood vessel and the chemical agents are injected into its vascular system after being washed with detergents	(i) The required pressure to drive the agent along the vascular system can cause the capillaries and small vessels to tea	Heart muscle, lung, liver, kidney, pancreas, small intestine, skeletal muscle, coronary artery	Tajima et al. (2020)
		(ii) The flow rate control is crucial		

Several authors have proposed the use of ECM synthesized during *in vitro* culture of primary cells, known as cell-derived ECMs (CD-ECMs) (Ha et al., 2020; Savitri et al., 2020). Among the advantages of this approach are the maintenance of the ECM architecture and its availability depending on the desired cell-line products, as well as the reduction in immunogenic response and infectious disease transmission. Similarly, some studies have demonstrated the secretion of different cytokines and growth factors by mesenchymal stem cells (MSCs) when cultured *in vitro*, such as bFGF, IGF-1 and VEGF, which are critical proteins for re-epithelialization and angiogenesis induction during wound healing (de Mayo et al., 2017; Becerra-Bayona et al., 2020). Although CD-ECMs may contain specific target biomolecules, they offer limited ability for tuning matrix properties to mimic those of native ECM. In contrast, dECM can be used in varying configurations (dressings, substitutes, powders and fillers), while closely resembling different aspects of natural ECM, such as mechanical properties, microstructure and composition, as evidenced by the content of key molecules: collagens (types I, II, III, IV, V, VI, VII, XIV), proteoglycans, fibrin, FN, vitronectin, versican, tenascin-C and growth factors (Tracy et al., 2016). In the context of diabetic foot ulcer treatments, dECM scaffolds act as a physiological reservoir of growth factors and cytokines that are involved in wound repair, providing signaling molecules that other treatments fail to supply, and improving cell migration and proliferation (Choi et al., 2013; Xu et al., 2021). In particular, decellularization of porcine small intestinal submucosa and urinary bladder matrix are among the most frequently used sources to recover native ECM. Therefore, various studies have reported the potential use of dECM-derived scaffolds as promising therapies for chronic wound healing (Table 3).

**TABLE 3:** Natural decellularized matrices for wound healing applications.

Decellularized source	Added molecules	Type of scaffold	*In vitro* studies	*In vivo* model	Time of *in vivo* studies	*In vivo* outcomes	Ref
Porcine dermis	HA	Dermal matrix	Not conducted	Rabbit full-thickness wounds	58 days	Contraction rate ↓ Collagen type I and III expression ↑	Zhao et al. (2013)
						Vascularization ↑	
Sheep dermis	HA and ADSCs	Dermal matrix used as covering dressing	Cell viability	Rat burns	4 weeks	Inflammation ↓	Alemzadeh et al. (2020)
						Angiogenesis ↑	
						Granulation tissue formation ↑	
						Wound closure ↑	
Mouse dermis	Chitosan	Membrane	Biostability Proliferation	Murine full-thickness wounds	28 days	Angiogenesis ↑	Lin et al. (2020)
	MSCs		Cell viability			Wound closure ↑	
			Cell adhesion			MSC retention ↑	
Rat dermis		Hydrogel		Rat full-thickness wounds	21 days	Angiogenesis ↑	Bankoti et al. (2020)
	Chitosan		Cell migration			Collagen deposition ↓	
	Carbon nanodots		Antibacterial properties			Wound closure ↑	
	MSCs		Cell viability			Re-epithelialization ↑	
						Epidermal junction formation ↑	
Porcine skin	Saccha-chitin	Hydrogel	Cell viability	Rat full-thickness wounds	14 days	Hair follicle growth ↑	Hsieh et al. (2020)
						Sweat gland formation ↑	
						Wound closure ↑	
						Collagen deposition ↑	
						Neovascularization ↑	
Porcine skin	None	Porous crosslinked membrane	Cell viability Cell adhesion	Rabbit full-thickness wounds	15 days	Wound closure ↑	Wang et al. (2021b)
						Inflammation ↓	
						Fibroblast migration ↑	
						Epidermal layer thickness ↓	
						Collagen organization ↑	
Porcine dermis	None	Crosslinked dermal matrix	Not conducted	Rat full-thickness wounds	90 days	Protease expression ↑	Carlson et al. (2013)
						Cellular and vascular infiltration ↓	
						Time of regeneration ↑	
Porcine dermis		Crosslinked dermal matrix	Cell viability	Rat full-thickness wounds	3 weeks	Degradation rate ↓	Chen et al. (2019)
	Chitosan		Enzymatic degradation			Antibacterial effect ↑	
	Silver nano-particles		Silver degradation			Wound closure ↑	
			Antibacterial properties				
Porcine dermis	Quercetin	Crosslinked Membrane	Biostability Antibacterial properties	Rat full-thickness wounds	3 weeks	Wound closure ↑	Wang et al. (2020a)
	Tea tree oil		Cell viability			Re-epithelialization ↑	
Porcine tissue		Porous crosslinked membrane	Cell viability	Not conducted	-	-	34,339,783
	Gelatin		Degradation				
	Chitosan		Antibacterial properties				
Goat small intestine submucosa	Curcumin	Crosslinked membrane	Degradation	Not conducted	-	-	Singh et al. (2022)
			Free radical scavenging tests				
			Cell viability				
			Hemo-compatibility assays				
Porcine urinary bladder		Membrane	Not conducted	Rat burns	28 days	ECM deposition ↑	Paramasivam et al. (2021)
						Granulation tissue formation ↑	
	pDNA-PDGF					Inflammation ↓	
	rBMSCs					Angiogenesis ↑	
						Wound closure ↑	
Porcine lung	None	Membrane	Not conducted	Rat-sub-cutaneous implantation	6 weeks	Cell infiltration ↑	Fernandez-Moure et al. (2016)
						Vascularization ↑	
Porcine adipose tissue	None	Hydrogel		Murine full-thickness wounds	14 days	Fibroblast migration ↑	Tan et al. (2019)
			Adipo-genesis			Wound closing ↑	
			Fibroblast migration			Epithelialization ↑	
						Angiogenesis ↑	
Human Adipose tissue	ADSCs	Hydrogel		Murine full-thickness wounds	14 days	Wound closure ↑	Chen et al. (2021)
			Cell Viability			Angiogenesis ↑	
			Cell proliferation			Skin appendages ↑	
			Angiogenic cytokines assay			Dermis thickness ↑	
Rat heart tissue	PLCL	Nano-fibrous membrane		Rat full-thickness wounds	3 weeks	Angiogenesis ↑	Kim et al. (2018)
			Cell proliferation			Scarring ↓	
			Cell adhesion			Granulation tissue ↑	
						Macrophage action ↑	
Equine pericardium	None	Crosslinked matrix	Not conducted	Murine full-thickness wounds	2 weeks	Re-epithelialization ↑	El Masry et al. (2019)
						Wound closure ↑	
						Collagen deposition ↑	
						Biofilm formation ↓	
Human placenta	None	Membrane	Not conducted	Rat Full-thickness wounds	4 weeks	Restoration of epidermis and dermis ↑	Choi et al. (2013)
						Wound closure ↑	
						Contraction rate ↓	
						Vascularization ↑	
Human umbilical cord Wharton’s jelly	None	Gelatinous material		Murine full-thickness wounds	7 days	Wound length ↓	Bakhtyar et al. (2017)
			Cell migration			Cell migration ↑	
			Cell viability			Cell differentiation ↑	
Human placenta, umbilical cord and amniotic membrane	None	Hydrogel		Murine full-thickness wounds	14 days	Wound closure ↑	Wang et al. (2021a)
			Cell migration			Skin appendage formation ↑	
			Cell proliferation			Pro-inflammatory gene expression ↓	
			Tube formation assay			Pro-angiogenic gene expression ↑	
Human Amnion	PRP	Membrane	Not conducted	Mouse burns	7 days	Epidermis differentiation ↑	Kshersagar et al. (2018)
						Keratinocyte proliferation ↑	
						Wound contraction ↓	
						Vascularization ↑	
Human amniotic membrane	None	Membrane	Not conducted	Rat full-thickness wound	8 months	Wound inflammation ↓	Song et al. (2017)
						Skin regeneration ↑	
						Scar formation ↓	
Human amniotic membrane	Silk fibroin	Electro-spun Membrane		Rabbit full-thickness ear wounds	50 days	Collagen deposition ↑	Gholipourmalekabadi et al. (2019)
			Cell viability			MMP1 deposition ↓	
			Cell adhesion			Scarring ↓	
Human amniotic membrane	None	Membrane	Cell viability	Mouse burns	15 days	Granulation tissue formation ↑	Milan et al. (2020)
						Angiogenesis ↑	
						Collagen maturation ↑	
Amniotic membrane		Membrane		Rabbit full-thickness burns	28 days	Epithelialization ↑	Ramakrishnan et al. (2020)
	Fibrin		Cell Adhesion			Angiogenesis ↑	
	HA		Cell proliferation			Skin appendage formation ↑	
Human Amniotic membrane	HWJMSCs	Membrane	Cell Viability	Rat burns	14 days	Re-epithelialization ↑	Hashemi et al. (2020)
						Granulation tissue formation ↑	
						Inflammation ↓	
Human amniotic membrane	None	Porous crosslinked membrane	Not conducted	Rat full-thickness wounds	21 days	Density of epidermal basal cells ↑	Nasiry et al. (2021)
						Length density of blood vessels ↑	
						Collagen deposition ↑	
						Gene expression related to regeneration ↑	
						Wound closure ↑	
Human amniotic membrane	Poly (1,8-octanediolco-citrate)	Membrane	Not conducted	Rat full-thickness muscle and back defects	2 weeks	Foreign body reaction ↓	Wang et al. (2020b)
				Rat liver injury		Inflammation ↓	
				Rat tibia defect		Fibrosis ↓	
Human amniotic membrane	Zinc oxide nano-particles derived from HAM proteins	Crosslinked Membrane	Antibacterial Assays	Not conducted	-	-	Ramasamy et al. (2021)
Bovine amniotic membrane	Chitosan	Sponge-like crosslinked membrane		Murine full-thickness wounds	14 days	Wound closure ↑	Yang et al. (2020)
			Cell Adhesion			Granulation tissue formation ↑	
			Cell proliferation			Angiogenesis ↑	
			Blood coagulation test			Fibroblast infiltration ↑	
			Cell viability			Sebaceous gland and hair follicle formation ↑	

AbbreviationsHA: hyaluronic acid; PRP: platelet rich plasma; PLCL: poly (l-lactide-co-caprolactone); ADSC: adipose derived stem cells; HWJMSCs: Human Wharton’s Jelly Mesenchymal Stem Cells; hSFs: Human Skin Fibroblasts; HAM: human amniotic membrane; PDGF: Platelet-derived Growth Factor; rBMSCs: Transfected Mesenchymal Stem Cells; ECM: extracellular matrix.

### Decellularized Extracellular Matrix Used as Membranes

#### Acellular Dermal Matrices

Acellular dermal matrices (ADMs) are considered artificial dermal substitutes that have been clinically utilized for treating chronic wounds, which are acellular full-thickness sections taken from donors, especially human cadavers or bovine/porcine skin, which are assessed for the presence of HIV, hepatitis, and syphilis (Dussoyer et al., 2020), and provide molecules that enhance cell attachment and neovascularization in wound surface repair (Wu et al., 2008). These features are usually enhanced by adding specific biological components to guide distinct cell responses within ADMs. For instance, Hsieh et al. optimized the decellularization of porcine skin with a formic acid treatment, and the resulted ADM was processed with pepsin to obtain hydrogels through pH and temperature control of the gelation process. These hydrogels were mixed with sacchachitin due to its chemotactic effect on inflammatory cells. The composite hydrogels promoted wound healing in full-thickness defects in rats after 14 days by facilitating angiogenesis, granulation tissue formation, and collagen deposition (Hsieh et al., 2020).

Similarly, the inclusion of exogenous hyaluronic acid (HA), which is commonly lost during decellularization, has shown the potential to modulate the production of collagen I and collagen III during ECM remodeling in wound healing (Zhao et al., 2013). In this sense, Zhoe et al. used a rabbit full-thickness wound model to observe the effect of HA-modified ADMs vs ADMs alone, showing that the former not only reduced scar formation and graft contraction because of its greater elasticity and anti-inflammatory properties, but also stimulated the expression of CD44 receptors, leading to a higher number of capillaries, compared to the ADM group. This may explain the therapeutic effects of HA on wound healing (Zhao et al., 2013). These findings are supported by the regulation of mRNA expression of cytokines during the inflammation phase, specifically, the downregulation of IL-1β and TGF-β1 as well as the upregulation of bFGF (Alemzadeh et al., 2020). In fact, overexpression of TGF-β1 may influence myofibroblast differentiation and cause prolonged acute inflammatory response, leading to excessive matrix deposition and exaggerated scar tissue formation (Jackson et al., 2012). That said, adequate coordination and progression of the phases in wound healing may be supported by the introduction of target motifs into ADMs.

Although it has been demonstrated that biologically modified ADMs have a more intensive action in skin repair, they inherently lack mechanical properties and have low resistance to enzymatic degradation, which limit their widespread use in the treatment of chronic wounds (Fernandez-Moure et al., 2016; Chen et al., 2019). The most common method to improve ADM mechanical strength is matrix crosslinking (Hu et al., 2013; Wang et al., 2021b; Xu et al., 2021), which may increase the time of tissue reconstruction, but provides longer-term structural integrity, thus, diminishing wound recurrence rate. In this context, Carlson et al. compared the expression of key genes involved in tissue regeneration when non-crosslinked human dermis and crosslinked porcine ADM were utilized to manage full-thickness wounds generated on rats (Carlson et al., 2013). The authors found that the crosslinked ADMs increased the expression of proteolytic enzymes (MMP-9 and CCL12) and lowered the ECM remodeling rate since cellular and vascular infiltration were reduced, relative to non-crosslinked ADMs.

Likewise, Chen at al. worked with naturally derived oxidized chitosan oligosaccharide (OCO), a biocompatible crosslinker, for the fixation of collagen on porcine ADMs through the formation of intra- and intermolecular bonds (Chen et al., 2019). Also, silver nanoparticles were loaded into the matrices to evaluate their antibacterial effect *in vitro,* prior to full-thickness wound studies in rats. The data established that OCO-crosslinked ADMs loaded with silver nanoparticles facilitated a more robust formation of the epidermis and granulation tissue, in comparison to the control, as shown by the higher expression of relevant factors for wound repair (bFGF, VEGF and PDGF). Similarly, other studies have demonstrated the advantage of combining natural antibacterial agents with ADMs, due to their low resistance to infection; besides, some crosslinking substances may reduce ADM biocompatibility and cause cytotoxicity (Zhu et al., 2016). To overcome these limitations, propolis and plant extracts, such as quercetin (QCT) and tea tree oil (TTO), have been proposed as alternative crosslinking agents that also provide antimicrobial properties (Sutjarittangtham et al., 2014). Specifically, TTO extracted from Melaleuca alternifolia is recognized by its antimicrobial activity (Reichling et al., 2006), while QCT has been reported to increase the tensile strength of porcine ADMs about three times, relative to pure ADM (Wang et al., 2020a). Their combined use for treating full-thickness wounds revealed accelerated wound repair due to the increase of re-epithelialization and wound closure rate. Therefore, crosslinking of ADMs may be required to produce wound dressings with superior mechanical strength and stability to ensure desired performance.

#### Acellular Decellularized Extracellular Matrix From Different Sources

Decellularized ECM-based scaffolds can be fabricated from various tissues and organs. For instance, porcine small intestine (SI) and urinary bladder (UB) are commercially used for skin reconstruction (OASIS, Table 1) (Paramasivam et al., 2021), although novel approaches are being studied to enhance biomolecule diversity within dECMs. The availability of cytokines, pro- and anti-inflammatory molecules as well as pro-angiogenic factors is crucial for properly guiding the four phases of normal skin regeneration. Therefore, among other tissues, heart-derived matrices are attractive since they preserve key molecules for wound healing, such as collagen, elastin, fibrillin, FN, and pro-angiogenic factors (ANG‐1, ANG‐3, CCN1, FGF‐1, FGF‐2, leptin, MMP‐9, NOV, SDF‐1, and VEGF‐β) (Seif-Naraghi et al., 2010; Kim et al., 2018). Masry et al. evaluated the role of equine pericardial collagen matrix (PCM) on wound healing progression, using a murine full-thickness model (El Masry et al., 2019). After 2 weeks, wound closure, re-epithelialization and collagen deposition increased compared to the control.

Moreover, PCMs induced and recruited phagocytic cells (macrophages) that lead to fibroblast differentiation into myofibroblasts, which produced collagen I to improve the tensile strength of the repaired skin, indicating an increase in the collagen type I:III ratio that supports formation of vascularized granulation tissue (Hurme et al., 1991; Tomasek et al., 2002). Indeed, the presence of Ang-1 in heart-derived dECM enhances both, blood vessel maturation and M2-macrophage phenotype differentiation, as reported by Kim et al., who assessed the influence of hybrid nanofibrous membranes fabricated by electrospinning of poly (l‐lactide‐co‐caprolactone) (PLCL) and rat heart dECM on rat full-thickness wound healing (Kim et al., 2018). The *in vitro* experiments confirmed the potential of the engineered membrane to produce huvec proliferation and angiogenesis. Also, the authors found denser vessel formation in the rats treated with the hybrid scaffolds, relative to the pure PLCL membranes. The results showed higher number of vessels positive for v-WF and alpha smooth muscle actin (α-SMA) factors, suggesting the capacity of heart-derived dECM to promote vasculogenesis and maturation of new vessels.

Furthermore, other promising sources for dECM production for wound healing applications are lung or adipose tissue-derived matrices (Fernandez-Moure et al., 2016; Tan et al., 2019; Chen et al., 2021). Acellular lung ECM (ALM) contains an association of collagen fibers along with a well-defined vascular architecture, which balance inflammatory response and support cell adhesion (Nichols et al., 2013). Fernandez-Moure et al. compared the ability of ADM vs ALM to stimulate angiogenesis and cellularization (Fernandez-Moure et al., 2016). Towards this, the ADM and ALM were subcutaneously implanted in rats, and after 6 weeks, much higher cell infiltration and vascularization were revealed in the ALM group, relative to the ADM treatment. Particularly, ALM-treated wounds displayed a higher number of infiltrated cells (1,663 ± 228 cells vs. 350 ± 230 cells per 500 μm^2^, respectively), as well as vessel ingrowth (57 ± 13 vessels vs. 9 ± 5 vessels per 1 mm^2^, respectively), compared to the ADM group. In the case of adipose tissue-derived matrices, adipogenesis is essential for wound healing progression, since adipose stem cells (ADSCs) produce various molecules that act as modulators in skin regeneration (Horsley and Watt, 2017; Plikus et al., 2017).

For instance, fibroblast migration increased when exposed to media obtained from adipocyte cultures, suggesting that adipocyte-secreted molecules may contribute to re-epithelialization and ECM remodeling during wound healing (Tan et al., 2019). Tan et al. examined the repair efficiency of adipose-derived dECM (ADECM) hydrogels with and without ADSCs in a murine full-thickness wound model. They found that both fibroblast migration and wound closing and epithelialization were more pronounced in the ADSC-loaded hydrogels, relative to their ADSC-free counterparts (Tan et al., 2019). Moreover, the data showed *in situ* induction of adipogenesis, which was evidenced by ADSC survival and higher number of adipocytes in the ADSC-loaded hydrogel treated group. Also, the results suggested a positive impact of ADSC-loaded ADCM hydrogels on skin appendage formation, such as hair follicle growth and blood vessel formation, data supported by increased VEGF expression by adipocytes. The paracrine activity of ADSCs was further investigated by Chen et al. by utilizing ADECM-derived hydrogels for delivering human ADSCs on murine full-thickness wounds (Chen et al., 2021). *In vitro* studies of ADSCs cultured on ADECM hydrogels showed significant secretion of angiogenic factors, including HGF (hepatocyte growth factor), VEGF, ANG (angiopoietin) and ANG-2, relative to ADSCs cultured on plastic surfaces. In addition to this, murine wounds had a faster closure rate and enhanced neovascularization when treated with ADSC-loaded ADECM hydrogels, compared to ADSC administration. Cumulatively, the results from using heart and adipose tissue-derived dECM indicate that their inherent architecture and biochemistry provide a favorable microenvironment for skin repair, which may be reinforced by incorporating ADSCs into the matrix.

Moreover, the role of specific pro-angiogenic factors (for example, VEGF, bFGF and PDGF) in the enhancement of the angiogenic response during wound healing has been elucidated. Nonetheless, their use is limited because of their short half-life, low availability, and high cost (Ehrbar et al., 2008). Thus, new engineered matrices modified with recombinant DNA, known as gene-activated matrices (GAMs), have been designed to include specific gene sequences of desirable growth factors to ensure their adequate expression (Pannier and Shea, 2004). In this context, taking advantage of the pro-angiogenic factor content in dECMs along with MSC regenerative properties, some genes have been introduced into dECMs to administer stem cells on impaired wounds, towards attaining an enhanced effect. For instance, Paramasivam et al. fabricated porcine UB with PDGF gene plasmid (Group C) or transfected MSCs with PDGF gene plasmid to embed them into UB dECMs (Group E) (Paramasivam et al., 2021). After 28 days of treating rat burns with the above approaches, acceleration of ECM deposition, granulation tissue formation, as well as angiogenesis and wound closure were observed in Group E, relative to Group C. These results may be explained by the chemo attractant action of PDGF over fibroblast migration and proliferation (Deuel et al., 1991). Similarly, PDGF may induce VEGF expression that leads to increased new vessel formation. Indeed, the authors reported higher VEGF expression and increased fibroblast proliferation in group E relative to Group C, which may have favored ECM deposition and vessel formation. Altogether, these findings indicate that dECM native features may be enhanced by combining them with stem cells and engineered DNA plasmids that promote relevant growth factor synthesis for controlled wound healing.

#### Acellular Decellularized Extracellular Matrix From Perinatal-Related Tissues

Perinatal tissues, such as placenta, umbilical cord, chorion, and amniotic membrane are associated with fetal development and discarded after birth. These tissues are appealing for wound healing applications because of their high content of molecules involved in skin regeneration. Notably, the human placenta ECM contains endogenous growth factors that provide a favorable niche for the proliferation and differentiation of cells, modulating injury repair (Choi et al., 2013). Similarly, a few studies have reported the application of Wharton’s jelly (WJ) for skin healing. WJ is a gelatinous substance that contains MSCs within a matrix that is mainly composed by collagen and HA, which secrete several molecules that may have an effect in diverse biological processes. In fact, many studies have reported the regeneration potential of both WJ-MSCs and their secretome by accelerating wound closure (Kamolz et al., 2014; Doi et al., 2016). In addition, the amniotic membrane, the inner layer of the placenta membrane that has a thickness between 0.02—5 mm and is composed of three layers (epithelial, basement and connective layers) (Zare-Bidaki et al., 2017; Milan et al., 2020), preserves a number of growth factors that participate in the progression of wound repair (Gholipourmalekabadi et al., 2016; Gholipourmalekabadi et al., 2019).

##### Acellular Decellularized Extracellular Matrix From Placenta and Wharton’s Jelly

The human placenta provides oxygen and nutrients to the fetus during pregnancy and eliminates waste products from its blood system. Decellularized placenta consists of various structural and adhesion proteins such as collagen (I, IV, VII, and XVII), elastin, LM, as well as proteoglycans, which serve as reservoir for many growth factors, including IGF, EGF, PDGF, FGF, VEGF and TGF-β (Choi et al., 2013; Singh et al., 2022). When dECM obtained from human placenta was used to treat rat full-thickness wounds, a well-structured basement membrane was deposited, and an increase in keratinocyte proliferation and differentiation was observed, which led to new epithelial tissue architecture that was very similar to original skin (Choi et al., 2013). Choi et al. produced dECM sheets from human placenta and observed adequate integration with the host’s wound tissue, in addition to superior expression of basal and epidermis layer markers (keratin 15 and loricrin), relative to the control (Choi et al., 2013).

Similarly, Wang et al. explored the effect of placenta-derived dECM (PL-dECM) hydrogels vs umbilical cord- and amniotic membrane-derived dECM (UC-dECM and AM-dECM, respectively) hydrogels in murine full-thickness wounds (Wang et al., 2021a). The results revealed different glycosaminoglycan content in each of the perinatal tissues, suggesting a distinct effect of each of the hydrogels on wound healing. In fact, PL-dECM hydrogels displayed a more potent effect on wound closure and skin appendage formation, compared to the other hydrogels. In particular, IL10 and TGF-β expression were upregulated, while IL6 and TNF-α expression were downregulated, which are anti- and pro-inflammatory cytokines, respectively. Also, M1-like macrophage differentiation was induced towards M2-macrophage phenotype by reducing the expression of iNOS and rising the expression of CD206 (M1 and M2 macrophage markers, respectively), relative to the other hydrogel formulations. In addition, PL-dECM hydrogels produced a better pro-angiogenic effect by enhancing expression of VEGF, ANG-1, FGF-2, and PDGF. In another study, Bakhtyar et al. employed WJ-dECM gels to better understand the role of some peptide growth factors that may accumulate in the WJ; for instance, IGF, FGF and TGF-β (Edmondson et al., 2003; Shalitin et al., 2003; Yu et al., 2003). After utilizing these dECM gels for treating murine full-thickness wounds for 7 days, the authors noticed the role of WJ-dECM on myofibroblast differentiation, diminishing wound healing time. Specifically, α-SMA expression (a myofibroblast marker) was higher in WJ-dECM treated groups vs the control, after 5 days of treatment (Bakhtyar et al., 2017), which suggested that WJ induced faster fibroblast differentiation toward myofibroblasts. In this context, components of PL- and WJ-dECMs play a role in inflammation, angiogenesis, and remodeling phases of wound healing, which may be employed to regulate specific responses to enhance restoration of impaired wounds.

##### Acellular Decellularized Extracellular Matrix From Amniotic Membrane

Due to its intrinsic regenerative properties, amniotic membrane (AM) is a promising tissue source for dECM therapeutic applications. During wound healing, decellularized human AM (dHAM) has been shown to modulate each of the healing stages by reducing inflammation, accelerating epithelialization, and preventing infection (Yuan et al., 2015). The observed restorative action of dHAM may be attributed, among other aspects, to its physical characteristics (e.g., durability, elasticity, permeability) and its ability to appropriately retain several growth factors. There are crucial components of dHAM that produce an anti-inflammatory and pro-angiogenic effect, being the most prominent factors EGF, KGF (keratinocyte growth factor), HGF, NGF (nerve growth factor), bFGF and TGF-β (Koob et al., 2015; Yuan et al., 2015; Milan et al., 2020). In this sense, Song et al. treated rat full-thickness wounds with dHAM and followed up on wound evolution for 8 months, observing a decrease in inflammation and scar formation (Song et al., 2017). These results were supported by the detected alterations of some growth factors; specifically, VEGF and α-SMA secretion were higher in dHAM-covered wounds, while TGF-β1 expression was reduced, in comparison to the control.

Likewise, when dHAM was used to cover mouse burns for 15 days, Milan et al. reported lower number of inflammatory cells as well as higher number of blood vessels, skin appendixes and collagen fibers on the granulation tissue of the dHAM group, relative to the control (Milan et al., 2020). In addition to applying pure dHAM, other authors have suggested the introduction of cells or other biological molecules to enhance the dHAM healing effect (Kshersagar et al., 2018; Hashemi et al., 2020; Ramakrishnan et al., 2020). For example, Hashemi et al. took advantage of the ability of MSCs to produce paracrine factors that accelerate injury repair (de Mayo et al., 2017) and explored the influence of dHAM combined with MSCs on rat burn repair (Hashemi et al., 2020). After 14 days of treatment, the data revealed significant differences in inflammation, re-epithelialization, and granulation tissue formation between rats treated with MSC-containing dHAM vs the group exposed to dHAM alone. Moreover, in the case of using exogenous factors, Kshersagar et al. proposed loading dHAM with platelet-rich plasma (PRP), since PRP contains several cytokines and growth factors (such as IFN-α, IL1, IL6, IL8, PDGF) that may alleviate inflammation during wound healing (Kshersagar et al., 2018). In a mouse burn model, the authors found that the PRP-loaded dHAM exhibited an enhancing effect on epidermis differentiation, keratinocyte proliferation, as well as wound contraction and vascularization. Collectively, these findings demonstrate the favorable microenvironment induced by dHAM for skin repair, while other studies have also reported dHAM ability to regulate the regeneration of other types of tissue injuries (Wang et al., 2020b).

Despite these promising results, dAM dressings have poor mechanical properties and lack long-term stability, which remains a concern for wound healing applications. Nevertheless, several studies have overcome these disadvantages by creating composite scaffolds (Gholipourmalekabadi et al., 2019; Yang et al., 2020; Nasiry et al., 2021). For instance, Yang et al. designed a bovine dAM-derived composite by utilizing a precursor and a crosslinking agent (chitosan and poly-ethylene glycol diglycidyl ether, respectively) to obtain a sponge-like membrane (CS-BAM) (Yang et al., 2020). The CS-BAM sponge exhibited stronger mechanical properties compared to the control and led to accelerated wound healing by simultaneously decreasing inflammation and increasing collagen deposition and skin appendix formation. Similarly, Gholipourmalekabadi et al. proposed the fabrication of a membrane composed of dHAM and electrospun silk fibroin (SF) to assess its efficacy on rabbit full-thickness ear wounds (Gholipourmalekabadi et al., 2019). They observed that the SF/HAM membrane modulated hypertrophic scar formation by reducing the expression of collagen I and upregulating the production of enzymes that participate in ECM remodeling. These results indicate that the balance between the synthesis of new ECM and ECM remodeling is crucial to avoid hypertrophic scar tissue formation.

In this context, Nasiry et al. suggested the use of a microporous crosslinked dHAM (MC-HAM) towards controlling ECM density during wound healing and avoiding limited cell migration and penetration into the site (Niknejad et al., 2008). The authors reported that healed rat full-thickness wounds improved their mechanical properties relative to the diabetic group; specifically, tensile strength, bending stiffness and energy absorption. In addition, after 21 days of MC-HAM scaffold engrafting, the number of neutrophils and macrophages significantly decreased, while the length density of blood vessels increased, data that was supported by the downregulation of pro-inflammatory factors (TNF-α and IL-1β) and the upregulation of pro-angiogenic genes (TGF-β, bFGF and VEGF). Cumulatively, this indicates that the fabrication of wound dressings derived from dHAM and exogenous cues that favor scaffold biostability and mechanical response, may be beneficial for properly guiding wound healing.

### Decellularized Extracellular Matrix Powders for Tissue Specific Regeneration Applications

ECM decellularization techniques are proving increasingly effective for the development of materials that can provide the biological stimuli present in the natural cell microenvironment, without the undesired risks associated with the use of xenogeneic or allogenic tissues. In this sense, the fabrication of tissue-specific scaffolds with biochemical and mechanical properties that more closely resemble the complexity of the native ECM has been one of the main research areas of focus (Storstein, 1989; Morris et al., 2018; Zahiri et al., 2018; Dongen et al., 2019; Ahmed et al., 2020; Hoang Thi et al., 2020; Park et al., 2020; Ventura et al., 2020; Liguori et al., 2021). Moreover, as the need to implement advanced manufacturing approaches such as 3D printing progressively becomes more compelling, enormous research efforts are being made to incorporate dECM into the design of functional bioinks to produce therapeutic alternatives for customized tissue regeneration applications.

#### Fabrication of Scaffolds for Tissue Regeneration

Recently, in the context of cardiovascular regeneration applications, Aquinas and coworkers explored the use of hydrogels derived from porcine cardiac dECM as material platforms for the controlled delivery of trophic factors produced by human ADSCs (Liguori et al., 2021). ADSCs were cultured for 48 h and the resulting conditioned medium was collected and further concentrated as follows: 20X, 200X, and 2000X. These concentrates were added to pepsin-digested dECM solutions as a 1/20 of the final volume to yield hydrogel precursors loaded with 1X, 10X, and 100X trophic factors, respectively. The release kinetics of seven pro-regenerative factors as well as four pro-inflammatory factors was studied for 5 days, revealing that the fabricated hydrogels provided a sustained release pattern over time. Nonetheless, it was also observed that pro-inflammatory factors were more abundantly released as the concentration of the conditioned medium in the hydrogel increased, which suggested that trophic factor binding kinetics within the hydrogel were affected by the concentration of the ADSC-derived conditioned medium.

In terms of wound healing applications, Van Dongen et al. prepared dECM from the adipose tissue of diabetic and non-diabetic human donors, and upon dECM digestion with pepsin, ADSC-conditioned culture medium was incorporated to create bioactive hydrogels for wound healing (Dongen et al., 2019). Taking advantage of the fact that ADSCs abundantly produce paracrine factors that influence ECM remodeling, the authors intended to explore the potential of the fabricated hydrogels to absorb and release these factors for the repair of fibroblast monolayers *in vitro*. Results indicated that the proposed hydrogels promoted fibroblast proliferation and migration, in addition to angiogenesis. Moreover, although no significant differences in terms of biological activity were found between the hydrogels from diabetic and non-diabetic origin, the latter exhibited greater mechanical stability and thus, proved to be more appropriate for clinical applications. In this way, the authors demonstrated the potential of ECM-derived hydrogels for the controlled delivery of allogenic paracrine factors and cytokines that provide biochemical support in wound repair processes.

Similarly, Jeon et al. developed a composite hydrogel from recombinant mussel adhesive proteins (MAP) and poly (N-isopropylacrylamide) (PNIPAM) for soft tissue regeneration applications (Jeon et al., 2020). Their goal was to harness the adhesive properties of MAP as well as the thermoresponsive nature of PNIPAM, a polymer that can undergo sol-gel transitions at physiological temperature, to create an injectable tissue-adhesive implant for the delivery of ADSCs and biochemical factors from decellularized adipose tissue (DAT) of human origin. Results from their *in vivo* tests indicated that the incorporation of DAT powder (5 wt%) into the hydrogel precursor significantly increased adipogenesis and vascularization, even in the absence of exogenous induction factors. Moreover, cumulative characterization data of the MAP-PNIPAM hydrogel alone proved its potential to be combined with other types of dECM powders for the delivery of tissue-specific biochemical stimuli.

Furthermore, Kuna and coworkers reported the development of a composite hydrogel system for skin regeneration, based on decellularized pig skin powder from galactose-⍺1,3-galactose knockout pigs, which was combined with HA (“PSG” sample) (Kuna et al., 2017). An additional formulation was prepared by adding human peripheral blood mononuclear cells (hPBMCs), to evaluate their effect on wound revascularization (“PSG-hPBMCs” sample). These two materials were tested *in vivo* for 25 days, including a pure HA control, using immuno-compromised mice with full-thickness skin wounds. The results indicated that, after 15 days of treatment, full wound closure was achieved in 66% of the mice treated with PSG and in 83% of the mice treated with PSG-hPBMCs, as opposed to the 0% observed for the HA controls. Nonetheless, at the end-point of the experiments (day 25), all mice displayed healed wounds, with some scarring observed in the HA control group. Moreover, enhanced wound healing and revascularization were attained in the PSG-hPBMCs group, as confirmed by CD31 staining. Thus, this composite hydrogel system proved its potential to be used as an autologous scaffold for successful skin tissue regeneration, without the need to incorporate stem cells, which are much harder to harvest in large numbers than PBMCs.

Moreover, regarding lung tissue repair, de Hilster and coworkers studied the fabrication of ECM-derived hydrogels from healthy and diseased human lung tissue (de Hilster et al., 2020). Tissue samples were decellularized following lyophilization and grinding to obtain an ECM powder, which was then enzymatically digested and gelled at 37°C. Stiffness measurements of the prepared hydrogels appeared to be within close range of those of their native tissue counterparts, although the viscoelastic response of the fabricated scaffolds was different from the corresponding tissue samples, since the latter exhibited less stress relaxation. Nonetheless, given that native lung tissue undergoes significant mechanical conditioning, these results revealed the possibility of creating ECM-derived scaffolds that provide biochemical and mechanical stimuli that mimic those naturally found in lung tissue, without the cell material that could trigger undesired immunological responses.

Finally, the development of effective ECM decellularization techniques has also a promising outlook for the advancement of therapeutic approaches in cartilage tissue engineering. For instance, the recent work conducted by Bordbar et al. addressed the evaluation and characterization of dECM hydrogels produced from sheep knee cartilage (Bordbar et al., 2020). Upon dECM digestion with pepsin and further neutralization, rabbit-derived mesenchymal stem cells (MSCs) were either encapsulated or seeded onto the resulting bioactive hydrogels, which were obtained by thermal gelation at 37°C. The experimental data showed that the proposed materials not only supported MSC adhesion and viability in 2D, but also promoted MSC proliferation and differentiation into chondrocytes in a 3D context. Along with this, the rheological properties of the hydrogel precursor indicated that the devised material could be successfully employed as an injectable hydrogel in minimally invasive therapies.

#### Design of Bio-Inks for Tissue Engineering and Disease Modeling

The use of dECM powder for tissue repair approaches has also extended to the additive manufacturing field, and thus, the development of bio-inks for 3D bioprinting. This technique allows for the fabrication of structures with highly controlled morphological features, which favors the ability to mimic the intricate architecture of native tissue as well as supports the advancement of customized tissue engineering therapies. Although widely used materials such as polyethylene glycol, alginate, and gelatin have been successfully used for the preparation of biocompatible inks (Ouyang et al., 2016; Ying et al., 2018), the incorporation of biochemical cues naturally found in the ECM is essential for guided tissue regeneration. In this sense, the ideal bio-ink should yield scaffolds that provide a 3D environment with biochemical and mechanical properties that resemble those of the target native tissue.

For instance, Kim and coworkers studied the fabrication of a bio-ink in which dECM powder from porcine liver was dispersed as microparticles (<100 µm) into a precursor mixture that contained gelatin, HA and fibrinogen (Kim et al., 2020). Prior to printing, the gelatin component was gelled at low temperatures, and once printing was completed, the bio-ink was further crosslinked by addition of thrombin. The resulting material was compared with a bio-ink counterpart prepared by the traditional method, in which the dECM powder was digested using pepsin, followed by printing and curing at 37°C. Characterization of all printed constructs revealed that the proposed bio-ink produced structures whose elastic modulus was up to 9.17 times higher than that of the material obtained with the traditional bio-ink, in addition to yielding much more consistent pore size and shape during the printing of 13 layers.

Furthermore, a lot of research efforts have been recently put into gaining a deeper understanding of the influence of several dECM processing variables on the material properties of the final bio-ink. For example, Zhao and coworkers evaluated the effect of dECM digestion time on the mechanical and biological properties of bio-inks prepared in the absence of any additional matrix materials (Zhao et al., 2020). To this end, powdered decellularized porcine tendon was subjected to different digestion times (3, 12, and 72 h) in acidic pepsin solution. At each time point, the pH of the solution was adjusted to 7.4, followed by rheological characterization. Furthermore, printability of the prepared inks was assessed by measuring extrudability, shape fidelity and layer stacking accuracy, using optical microscopy and digital imaging techniques. The authors were able to demonstrate that partially digested dECM (3 h) yielded an ink precursor with desirable viscosity and printability, as well as higher construct reproducibility up to 30 layers, relative to the ink obtained after 72 h of digestion. All of this was achieved while maintaining the viability levels of bone marrow MSCs above 85%. Finally, it was also demonstrated that the bioprinted scaffolds supported significant MSC differentiation towards tendon tissue, as evidenced by COL1A1, EGR1, TNC, and TNMD PCR analysis.

Later, Zhao et al. also studied the impact of the type of dECM digestion solution on the mechanical properties and cytocompatibility of hydrogels produced from tendon dECM derived bio-inks (Zhao et al., 2021). They examined three different acidic solutions for the preparation of pepsin-based digestion solutions: 0.5 M acetic acid, 0.1 M HCl and 0.02 M HCl. Upon neutralization of the dECM solutions, bio-ink precursors were obtained. The osmotic pressure of the bio-ink from the acetic acid solution could not be adjusted to physiological levels, which in turn translated into high cytotoxicity, as evidenced by viability tests conducted on encapsulated bone marrow MSCs, prior to 3D printing. Moreover, the authors found that the mechanical properties of the 3D printed constructs decreased as the acid concentration in the digestion solution increased, being the softer hydrogels more inducing of a tendon-like phenotype.

In line with the abovementioned studies, Jeong and coworkers studied the decellularization efficiency of different types of detergents on porcine liver tissue, as well as their effect on the cytocompatibility, printability, and biochemical and mechanical properties of the resulting dECM derived bio-inks (Jeong et al., 2021). Sodium dodecyl sulfate (SDS), sodium deoxycholate (SDC), Triton X-100 (TX), and Triton X-100 with ammonium hydroxide (TXA) were examined. The authors found that, while SDS appeared to be the most efficient for DNA removal (up to 94% removal in 24 h), about 98% of GAGs and elastin from the native ECM was lost during decellularization with this detergent. On the contrary, TXA dECM samples exhibited adequate preservation of GAGs, collagen, and elastin, in addition to the fastest gelation rate and thus, the best printability. Moreover, TXA treated tissue samples yielded the dECM bio-ink with the most robust mechanical properties, because of its highly organized and packed fibrillar structure, as evidenced by SEM imaging results. This, together with the favorable cytocompatibility observed for the TXA group, demonstrated the suitability of TXA for the fabrication of liver dECM bio-inks.

Moreover, regarding skin dECM bio-inks, Jorgensen et al. examined the effect of the presence of human skin dECM on the bioactivity and mechanical properties of fibrinogen hydrogels for skin bioprinting applications (Jorgensen et al., 2020). Once the corresponding dECM powder was obtained, it was pepsin-solubilized at different concentrations, and these solutions were then combined with the bovine fibrinogen hydrogel precursor at a 1:1 volumetric ratio. Human skin fibroblasts were encapsulated in these hydrogels, following by crosslinking with 20 μL/ml thrombin. After a 2-weeks *in vitro* culture, the fibrinogen hydrogels containing dECM showed enhanced cellularity and almost a 50% increase in cell viability, relative to the pure fibrinogen counterparts. Also, rheological and printability characterization revealed that the proposed hydrogels exhibited favorable shear-thinning properties for the reproducible fabrication of bioprinted constructs.

Similarly, Lee et al. examined the biocompatibility of a bio-ink composed of dECM powder from porcine dermis dissolved in a 2–3% sodium alginate hydrogel, at 10 and 20 mg/ml (Lee et al., 2020). Rheological characterization of the prepared inks showed that ink viscosity could be tuned by varying dECM powder concentration. Moreover, mouse embryonic fibroblasts (NIH3T3) were mixed with the hydrogels at 1 × 10^6^ cells/mL prior to 3D printing, and once the scaffold was printed, it was further crosslinked in 5% CaCl_2_. After 1 week of culture, live/dead staining and cell metabolic activity tests indicated that cell viability levels within the constructs were maintained at around 75%.

Another remarkable example of skin dECM bio-ink development has been recently shown by Won and coworkers (Won et al., 2019). In their experiments, porcine dermal skin was decellularized to produce collagen, elastin, and GAG-rich dECM powder, which was then pepsin-digested, neutralized, and further combined with a suspension of human dermal fibroblasts. The cell-loaded bio-ink was used for the bioprinting of constructs that were cultured for 7 and 14 days at 37°C. The authors were able to show that the printed 3D dECM constructs maintained their shape, favored cell viability levels of at least 90%, and supported normal fibroblast cell shape during their first week of culture. In addition to this, microarray analyses performed after 1 and 2 weeks of culture revealed that the dECM constructs induced significantly higher upregulation of genes related to dermis and epidermis formation, relative to their corresponding collagen controls.

For their part, Falcones et al. examined the feasibility of using lung dECM bio-inks for enhancing the therapeutic effect of MSCs, in the context of MSC-based therapies for the treatment of respiratory diseases (Falcones et al., 2021). Specifically, the authors employed porcine lung dECM to produce bio-inks within which lung resident MSCs were embedded. Following this, 3D constructs were bioprinted and cultured *in vitro* for 7 days, with the purpose of pre-conditioning the MSCs. After this, MSCs were released from the constructs through collagenase digestion, recovered, and further used for direct co-culture with human primary small airway epithelial cells (HSAECs) in the presence of lipopolysaccharides (LPS), to create an *in vitro* model of acute respiratory distress syndrome. The authors were able to show that the fabricated lung dECM 3D constructs provided a biochemical and biomechanical environment that promoted desirable MSC morphology and adhesion properties, in addition to improved immunomodulatory potential, as evidenced by the higher expression of CXCR4 and the lower production of IL6, relative to MSCs that were pre-conditioned by standard 2D culture in tissue culture polystyrene.

Moreover, it is noteworthy that dECM derived bio-inks have not only been used for the design of 3D constructs for tissue regeneration, but also for the development of disease models. Specifically, Kort-Mascort et al. recently created a dECM bio-ink that was mechanically reinforced with alginate and gelatin for the reproducible bioprinting of hydrogels that mimic head and neck tumor microenvironments (Kort-Mascort et al., 2021). Towards this end, porcine tongue was selected as the dECM source since this is one of the major sites for head and neck squamous cell carcinoma (HNSCC) development. Prior to bioprinting, human HNSCC cells were encapsulated in these bio-inks. As a result, composite 3D constructs with enhanced printability and mechanical properties that resembled those of native tumors were obtained. After 19 days of *in vitro* culture, cell viability in these hydrogels remained at around 95%, while favorable cell proliferation and tumor formation were evidenced by keratin expression. Finally, the designed tumor model was successfully used for *in vitro* chemotherapy studies using cisplatin and 5-fluorouracil, two drugs clinically employed to treat head and neck cancer.

Finally, Kim and coworkers developed bio-inks from porcine gastric tissue for the creation of *in vitro* 3D gastric cancer models. Prior to bioprinting, human gastric cancer cells (AGS, SNU-1, and KATO3 cell lines) were encapsulated in the bio-ink precursors, and cellulose nanoparticles were incorporated as a mechanical reinforcement agent (Kim et al., 2021). The resulting 3D constructs displayed mechanical properties that were physiologically relevant, and supported the enhanced expression of MMP2, β-catenin, and integrin β1, relative to their collagen and Matrigel controls. These markers are associated with the aggressive and drug-resistant nature of gastric cancer. Cumulatively, the results from the abovementioned studies demonstrate the promising potential of combining additive manufacturing techniques with dECM powders to produce *in vitro* 3D platforms that can closely resemble *in vivo* human microenvironments and, thus, could be used as reliable tools for either tissue regeneration, or drug testing pre-clinical studies in the absence of animal models.

## Discussion

Wound healing is a complex multi-step process that involves soluble factors, blood elements, and cells, which interact synergistically in an ECM, through mechanisms of dynamic reciprocity, where wound molecules interact with cell surface receptors and trigger intracellular signal transduction cascades, promoting expression of specific genes and secretion of signal molecules, whose in turn, coordinate the wound healing stages (Bhat and Bissell, 2014; Rousselle et al., 2019). Nevertheless, based on the type of tissue, healing is a process with distinct features. In the case of normal skin, there are three layers that intervene in the process: the epidermis, or the outer layer, which contains the sebaceous glands, sweat glands, and hair follicles; the dermis, which contains a rich ECM that provides strength, nutrients, and immunity; and finally, the hypodermis, which is the subcutaneous adipose tissue that functions as an energy reserve (Gurtner et al., 2008). Despite the differences among these layers, each of their ECM contains structural proteins such as FN, HA, proteoglycans, collagens, glycosaminoglycans, and elastin, in addition to non-structural matricellular proteins, including secreted protein acidic and rich in cysteine (SPARC), tenascin-C, osteopontin, and thrombospondins. The presence and characteristics of these molecules are dynamic during wound healing, and even under non-healing (normal) conditions, they are continuously being regenerated (Takeo et al., 2015; Rousselle et al., 2019).

Even though there is a number of commercially available skin substitutes and tissue-engineered skin products to manage impaired wounds, high manufacturing costs, excessive inflammation, as well as disease transmission have made their massive implementation difficult. To surmount these barriers, the use of dECM obtained from different tissues has emerged as a promising approach since they provide a favorable regeneration microenvironment by preserving cytokines, proteins and growth factors from the natural ECM, which altogether coordinate inflammation, re-epithelialization and ECM remodeling processes (Choi et al., 2013). In fact, as described in previous sections, several studies have elucidated the role of relevant molecules such as TNF-α, TGF-β, PDGF, VEGF, IGF-1, and HGF in wound healing, and have demonstrated their presence in acellular tissues derived from dermis, UB, SI, heart, adipose, and perinatal membranes (Table 3). Also, it is well known that different types of cells play a critical role. For instance, fibroblasts produce numerous ECM components, including collagens, FN, and proteoglycans (Tracy et al., 2016) that regulate the balance between degradation and synthesis of new skin. Thus, several researchers have developed dECM-engineered scaffolds that have the ability to modulate the wound healing response to avoid long-term inflammation and hypertrophic scar tissue formation (Table 3) (Biernacka et al., 2011).

To gain insight into the role of ECM elements and microenvironment on activation of signaling cascades related to wound healing processes, some studies have examined the individual effect of proteins and growth factors using *in vivo* models (Yang et al., 2015; Castellanos et al., 2017; Pozzi et al., 2017). Regarding cytokines, the literature reports that TGF-β and bFGF are pleiotropic factors that favor multiple wound-related processes, such as fibroblast and keratinocyte migration, fibroblast differentiation into myofibroblast, M1-like macrophage phenotype towards M2-like phenotype, as well as collagen deposition and remodeling (Castellanos et al., 2017). Similarly, other researchers have suggested the importance of GAG content on dECM because sulfated components may influence the availability of growth factors involved in angiogenesis and vasculogenesis during wound healing (Pozzi et al., 2017). In addition to this, a growing body of evidence indicates the enhanced regenerative effect that may derive from the interaction between the skin and surrounding tissues, such as adipogenic tissue. In particular, adipogenesis plays three key roles: 1) expression of adipogenic genes that trigger skin-related repair; 2) secretion of molecules by adipocytes; and 3) provision of the adipogenic microenvironment that leads to skin appendage formation (Yang et al., 2015). Therefore, natural matrices are promising platforms for the advancement of engineered scaffolds that effectively regulate and promote specific tissue regeneration responses, such as those involved in wound healing processes.
